# Detection of zoonotic protozoa in raccoons (*Procyon lotor*) from aquaculture zones in Saxony (Germany): One health perspective

**DOI:** 10.1016/j.onehlt.2026.101477

**Published:** 2026-06-13

**Authors:** Zoё Tess Lara Lindhorst, Maria Sophia Unterköfler, Piotr Solarczyk, Michael Striese, Diana Jeschke, Elisabeth Striese, Hermann Ansorge, Hans-Peter Fuehrer, Mike Heddergott

**Affiliations:** a Institute of Parasitology, Department of Biological Sciences and Pathobiology, University of Veterinary Medicine Vienna, Austria; b Department of Biology and Medical Parasitology, Poznan University of Medical Sciences, Poznan, Poland; c Lutra, Büro für Naturschutz und Landschaftsökologische Forschung, Boxberg, Germany; d Senckenberg Museum of Natural History Görlitz, Germany; e Federal Research Institute for Animal Health, Friedrich-Loeffler-Institut, Greifswald-Insel Riems, Germany; f International Institute Zittau, Technische Universität Dresden, Zittau, Germany; g Department of Zoology, Musée National d'Histoire Naturelle, Luxembourg

**Keywords:** Raccoon, *Giardia duodenalis* sub-assemblage BIV, *Cryptosporidium* sp. skunk genotype, Zoonosis, Water-borne pathogens, Germany

## Abstract

The raccoon (*Procyon lotor*), an invasive species in Europe, is a potential reservoir for zoonotic pathogens, posing risks to human, animal, and environmental health. This study investigates the prevalence and genetic diversity of *Giardia duodenalis* and *Cryptosporidium* spp. in raccoons from the Upper Lusatian Heath and Pond Landscape (Saxony, Germany), adopting a One Health perspective at the wildlife–aquaculture interface. Fecal samples (*n* = 104) were collected from culled raccoons (2020−2022) across six pond farming areas. Samples were screened using rapid immunochromatographic antigen tests, followed by molecular characterization via PCR and sequencing of specific loci (*gdh*, *tpi*, and *bg* for *Giardia*; *SSU rRNA* and *gp60* for *Cryptosporidium*). *Giardia duodenalis* assemblage B (sub-assemblage BIV) was detected in 24% of samples. Significant associations were observed with location (*p* = 0.038) and aquaculture company (*p* = 0.028). *Cryptosporidium* sp. skunk genotype was identified in 2% of samples, showing no significant correlation with the tested parameters. The identification of *G. duodenalis* (BIV) and *Cryptosporidium* skunk genotype highlights the role of raccoons as potential reservoirs in water-rich production landscapes. The high prevalence of *Giardia* and its spatial heterogeneity suggest that site-specific pond management and raccoon dynamics might influence pathogen loads. This study provides the first molecular evidence of zoonotic protozoa in raccoons within this specialized aquaculture context. The results underscore the necessity of integrating wildlife monitoring into aquaculture management. Aligning invasive species control with One Health surveillance is essential to mitigate zoonotic risks and safeguard public health in aquatic ecosystems.

## Highlights

• First detection of *Giardia duodenalis* BIV in raccoons from Saxony, Germany.
• Detection of zoonotic *Cryptosporidium* skunk genotype (*C*SkG) in raccoons.
• *Giardia duodenalis* correlated with location and aquaculture company.
• Highlights one health's role in addressing zoonotic risks in aquaculture zones.

## Graphical abstract

**Figure:**
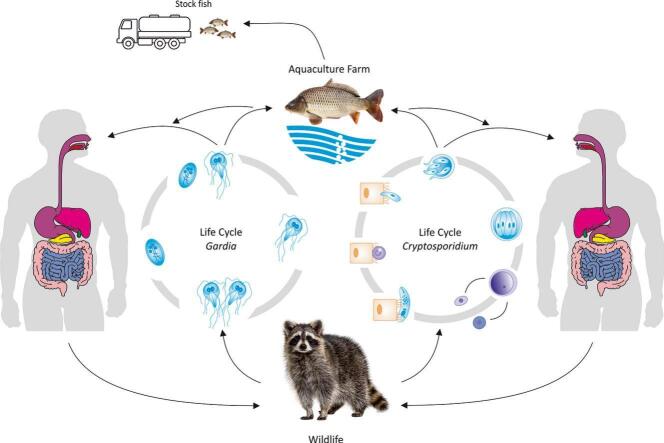

## 1 Introduction

Aquaculture—the controlled breeding of aquatic organisms—is one of the fastest growing sectors of global food production. As such, it has become a key factor in food security in light of population growth and rising demand for protein-rich foods [1], [2]. Aquaculture systems are generally divided into closed systems, which are largely isolated from external influences, and open systems, which interact directly with their environment and are therefore exposed to wildlife, water quality, and climate fluctuations [3].

The Upper Lusatian Heath and Pond Landscape (Saxony, Germany) represents Europe's largest commercially managed pond system [4]. Dominating the sector, common carp (*Cyprinus carpio*) accounted for 1665 tons in 2024, with 90% produced in open systems and 41% of the total yield concentrated in the Görlitz district [5]. Another important economic component of aquaculture operations in this region is the breeding and rearing of young fish, which are marketed throughout Germany as stock fish for fishing waters [6].

*Giardia duodenalis* (syn.: *G. intestinalis* and *G. lamblia*) and *Cryptosporidium* spp. are globally distributed protozoa causing diarrheal diseases in various hosts, including humans [7]. Due to their public health impact, they are included in the WHO Neglected Diseases Initiative [8]. While human cryptosporidiosis represents a leading cause of mortality among protozoan-induced diarrheal diseases worldwide [9], giardiasis is predominantly self-limiting and seldom fatal in humans. However, it remains a substantial clinical challenge due to its potential for reinfections [10]. Currently, 44 *Cryptosporidium* species and more than 120 genotypes are recognized, of which 19 species and four genotypes have been detected in humans [7]. In Europe, at least eleven *Cryptosporidium* species have been identified in wild carnivores over the past 20 years, with *Cryptosporidium* sp. skunk genotype, *C. canis*, and *C. ditrichi* being the most common [11]. G*iardia duodenalis* is a species complex comprising eight distinct genotypes, designated assemblages A to H. Within this complex, assemblages A and B (particularly sub-assemblages AII and BIV) are considered to possess the highest zoonotic potential due to their broad host spectrum [12]. In Europe, these two assemblages have been detected in seven different carnivore species [11]. Although infections with *Cryptosporidium* and *Giardia* in wild carnivores are generally considered asymptomatic, only limited data are available regarding their epidemiology and clinical significance in wildlife [11].

The raccoon (*Procyon lotor*) is a carnivore native to North and Central America. It has spread throughout parts of Asia and Europe, and is now one of the four most widely distributed invasive mammal species in Europe [13]. In Germany, populations have increased significantly since their introduction, with well-established populations identified in northeastern regions, such as Saxony [14]. Their success is largely due to their high ecological plasticity, omnivorous diet, and behavioral adaptability, which allow them to thrive in both urban and rural environments [15]. As a semi-aquatic species, raccoons show a strong affinity for bodies of water such as streams, lakes, and wetlands [16]. In areas where they spread, this dispersal preference promotes interactions with aquatic ecosystems and the wildlife found there, raising concerns about ecological impacts and the introduction or spread of pathogens [17].

The presence of zoonotic protozoa in aquaculture areas is an important concern in terms of the One Health concept, given the risk of environmental contamination and waterborne transmission at the human–animal–environment interface. While the raccoon has recently been confirmed as a carrier of *G. duodenalis* and *Cryptosporidium* spp. in Germany [18], [19], [20], its role in specific water-rich landscapes remains understudied. Against this background, the present study investigates the prevalence, genetic diversity, and potential ecological impact of these two protozoa in raccoons from aquaculture areas in eastern Saxony. By combining molecular analyses with the ecological context, this research aims to shed light on the role of raccoons as potential reservoir hosts, clarify their contribution to zoonotic risks in water-rich landscapes, and gain insights for management strategies to combat invasive species and aquaculture-related diseases.

## 2 Material and methods

### 2.1 Ethical statement

The raccoon is an invasive species in Germany. It is not protected by law and can be hunted by licensed hunters [21], [22]. On the premises of the aquaculture farms examined in this study, hunting is carried out as part of a predator management program on behalf of the Saxon district of Görlitz. All animals were killed legally and made available to the authors for this study. No animals were killed specifically for this study.

### 2.2 Areas of study

**Fig. 1:**
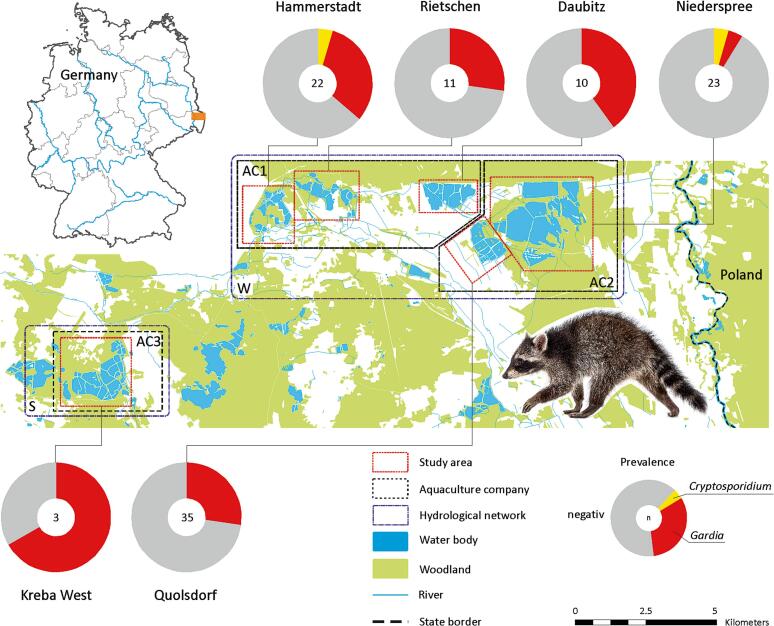
Map of studied area, aquaculture companies (AC) and water supply companies (S and W) in the Upper Lusatian Heath and Pond landscape in Saxony in the northern district of Görlitz (eastern Germany). The pie charts show the prevalence (in %) of wild raccoons (*Procyon lotor*) infected with *Gardia duodenalis* and *Cryptosporidium* skunk genotype (*C*SkG) as well as the sample size (n) of the individual study areas. Abbreviations: AC1 = Rietschen; AC2 = Kreba-Fisch; AC 3 = Teichwirtschaft Kreba; S = Schwarzer Schöps; W = Weißer Schöps.

The raccoons examined in this study originate from six pond farming areas in the Upper Lusatian Heath and Pond landscape in the northern part of the Görlitz district in Saxony: Daubitz (*n* = 10), Hammerstadt (*n* = 22), Kreba West (*n* = 3), Niederspree (*n* = 23), Quolsdorf (*n* = 35), and Rietschen (*n* = 11) (Fig. 1). These locations are associated with three aquaculture companies (AC). The pond farming areas of Daubitz, Hammerstadt, and Rietschen are managed by Fischzucht Rietschen (AC1), those in Niederspree and Quolsdorf by Kreba-Fisch GmbH (AC2), and the Kreba West area by Teichwirtschaft Kreba (AC3) (Fig. 1). Hydrologically, the region belongs to the Spree river basin, which drains into the North Sea via the Elbe River, with the main waterways being the Weißer Schöps and Schwarzer Schöps rivers. The study areas are characterized by an interconnected system of more than 1000 ponds, where water supply and drainage are managed through a complex network of ditches and weirs.

### 2.3 Sample collection

Between June 2020 and November 2022, fecal samples were collected from 104 raccoons—representing 50% of all animals culled in the study areas during the investigation period. Immediately after being killed, the carcasses were frozen with proof of origin and sent to the Senckenberg Museum in Görlitz within a week. Here, necropsies were performed to continue the ongoing monitoring of the occurrence and surveillance of *Baylisascaris procyonis* in Germany [23], [24]. During necropsy, 5–10 g of feces were taken from the rectum and stored in 70% ethanol at −20 °C until further analysis at the University of Veterinary Medicine Vienna. The sex of each raccoon was determined. The age of each animal was determined based on tooth wear [25]. The animals were divided into two age groups: adult (>1 year) and juvenile (≤1 year). The raccoons examined in this study were also the subject of earlier parasitological studies [26], [27].

### 2.4 Antigen tests

All samples were screened using a rapid immunochromatographic assay for *G. duodenalis* and *Cryptosporidium* spp. (FASTest® CRYPTO-GIARDIA Strip, MegaCor) following the manufacturer's instructions. While this assay is validated for a variety of domestic and wild animals, its application for raccoons represents an off-label use in a wildlife context.

### 2.5 DNA extraction

For reasons of cost-effectiveness, DNA extraction was performed exclusively on samples that yielded positive results in the initial antigen screening. DNA was extracted using the NucleoSpin® Soil Mini Kit (MACHEREY-NAGEL) according to the manufacturer's instructions.

### 2.6 PCR amplification and sequencing

*Giardia duodenalis* was targeted at the *gdh*, *tpi*, and *bg* loci (Table 1, Table A.1), while *Cryptosporidium* spp. was analyzed using the 18S *SSU rRNA* and *gp60* genes (Table 2, Table A.2). PCRs included positive and negative controls and were performed using GoTaq™ DNA Polymerase (Promega, USA). Products were visualized on 1.8% agarose gels with Midori Green (NIPPON Genetics). Positive samples were sequenced (Microsynth), and sequences were edited in BioEdit v.5.0.9 before NCBI database comparison.

**Table 1:** Oligonucleotide sequences of *Giardia duodenalis* primers used in the present study.

Genetic marker	Nest	Primer sequences (5′ ➔ 3′)	Product size (bp)	Annealing temperature	Reference
*gdh*	N1	gdh_1F: TTCCGTRTYCAGTACAACTC	754	60 °C	[28], [29]
		gdh_2R: CCTCGTYCTGRGTGGCGCA			
	N2	gdh_3F: ATGACYGAGCTYCAGAGGCACGT	530	58 °C	
		gdh_4R: GTGGCGCARGGCATGATGCA			
*tpi*	N1	AL3543-for: AAATIATGCCTGCTCGTCG	605	50 °C	[30], [31]
		AL3546-rev: CAAACCTTITCCGCAAACC			
	N2	AL3544-for: CCCTTCATCGGIGGTAACTT	530	50 °C	
		AL3545-rev: GTGGCCACCACICCCGTGCC			
*bg*	N1	G7: AAGCCCGACGACCTCACCCGCAGTGC		65 °C	[32]
		G759: GAGGCCGCCCTGGATCTTCGAGACGAC			
	N2	β-GIAR2-F: GAACGAGATCGAGGTCCG	511	55 °C	
		β-GIAR2-R: CTCGACGAGCTTCGTGTT

**Table 2:** Oligonucleotide sequences of *Cryptosporidium* spp. primers used in the present study.

Genetic marker	Nest	Primer sequences (5′ ➔ 3′)	Product size (bp)	Annealing temperature	Reference
18S *SSU rRNA*	N1	18SiCF2: GACATATCATTCAAGTTTCTGACC	763	58 °C	[33]
		18SiCR2: CTGAAGGAGTAAGGAACAACC			
	N2	18SiCF1: CCTATCAGCTTTAGACGGTAGG	587	58 °C	
		18SiCR1: TCTAAGAATTTCACCTCTGACTG			
*gp60*	N1	AL-3531_fw: ATAGTCTCCGCTGTATTC	879	59 °C	[34]
		AL-3535_rev: GGAAGGAACGATGTATCT			
	N2	AL-3532_fw: TCCGCTGTATTCTCAGCC	846	50 °C	
		AL-3534_rev: GCAGAGGAACCAGCATC			

### 2.7 Phylogenetic analysis

For *G. duodenalis*, a phylogenetic analysis at the *tpi* locus was performed using one reference sequence for each assemblage, according to Sprong et al. [35]. For assemblages A and B, sub-assemblage sequences were also included, while assemblage H was excluded due to the absence of a reference at the *tpi* locus (Table B.1). The alignment (490 bp) was trimmed to 430 bp using TrimAl v.1.3 [36] and rooted with *Giardia ardeae* (AF069564). Maximum likelihood (ML; 1000 replicates) and Bayesian inference (BI) trees were generated using W-IQ-TREE [37] and MrBayes v.3.2.7 [38] (GTR + G/G4 models). BI was run for 10^6^ generations with a 25% burn-in. The final tree was visualized in CorelDRAW 2024.

### 2.8 Statistical analysis

Statistical analyses were performed using R version 4.3.2 (R Foundation for Statistical Computing) to assess associations between pathogen occurrence and host factors (sample origin (year and location), responsible aquaculture company, water supply, sex, and age). Only samples that tested positive for *G. duodenalis* and *Cryptosporidium* spp., based on sequencing of the *tpi* genetic locus and the *SSU rRNA* gene, respectively, were included in the correlation analysis. Depending on cell frequencies, either the Pearson's chi-squared test (*χ*^2^) or the Fisher's exact test was applied. Statistical significance was defined as *P* < 0.05. The 95% confidence intervals (CI) for proportions were calculated using the Clopper-Pearson exact method.

## 3 Results

### 3.1 Occurrence and molecular characterization of Giardia duodenalis

Of 104 samples tested, the *Giardia* antigen test yielded 26 (25.0%; 95% CI: 17.0–34.5) positive and 78 (75.0%; 95% CI: 65.6–83.0) negative results. In the PCR assay targeting the *tpi* gene locus, 25 samples (24.0%; 95% CI: 16.2–33.4) tested positive, whereas all samples remained negative at the *gdh* and *bg* loci.

**Fig. 2:**
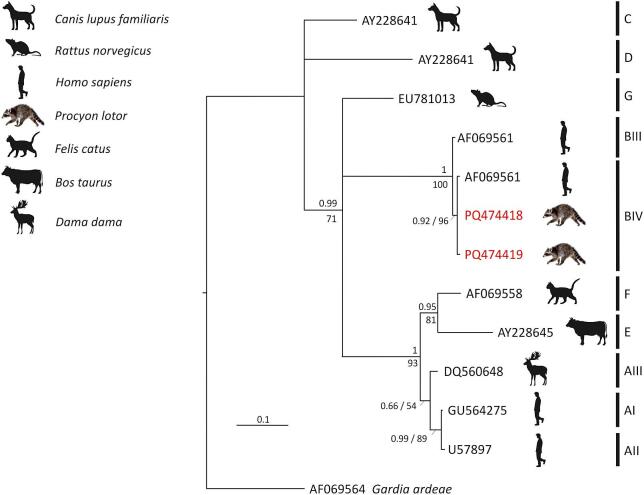
Bayesian Inference tree based on *tpi* (430 nucleotide positions) sequences of *Giardia duodenalis*. Reference sequences were chosen based on Sprong et al. [35]. Additional information on references can be found in Table B.1. Assemblage H was not included, because, to the authors' knowledge, it has not yet been uploaded to GenBank on the *tpi* genetic locus. *Giardia ardeae* was used as outgroup. Nodes are marked with Bayesian posterior probabilities and Maximum Likelihood bootstrap values. Assemblage, accession number and host are provided for every sequence. In the case of Assemblage A and B, sub-assemblages are also provided. Sequences that are marked in red were obtained in this study. The scale bar indicates the expected mean number of substitutions per site according to the model of sequence evolution applied. (For interpretation of the references to colour in this figure legend, the reader is referred to the web version of this article.)

All sequences obtained showed the highest similarity to *G. duodenalis* assemblage B, matching a reference sequence previously reported from a human host in Canada (GenBank: KM190823). Eighteen sequences identical to the reference (100% identity and query coverage) were deposited in GenBank under accession numbers PQ474418 and PV976788–PV976804. Six additional identical sequences were excluded from submission due to incomplete alignment (forward reads only). One sequence variant, differing at positions 223 and 224 (99.6% identity), was deposited under accession number PQ474419. Phylogenetic analysis showed that both haplotypes clustered within sub-assemblage BIV (Fig. 2).

No statistically significant correlations were found between *Giardia* positivity and year of death (*χ*^2^ = 0.903, *df* = 2, *p* = 0.204), water supply (*p* = 0.142, OR = 0.151, 95% CI: 0.002–3.020), sex (*χ*^2^ = 0.110, *df* = 1, *p* = 0.740), or age (*χ*^2^ = 1.439, *df* = 1, *p* = 0.230) (Table C.1). In contrast, *Giardia* positivity was significantly associated with the location (*p* = 0.038) and the responsible aquaculture company (*p* = 0.028) (Fig. 1; Table 3).

**Table 3:** Significant associations in *Giardia* positivity by Location of death and aquaculture company in raccoons from Saxony.

Category	n/N	Proportion (%)	95% CI Lower (%)	95% CI Upper (%)
Location of death				
Daubitz	4/10	40.0	12.2	73.8
Hammerstadt	7/22	31.8	13.9	54.9
Kreba West	2/3	66.7	9.4	99.2
Niederspree	1/23	4.3	0.1	22.0
Quolsdorf	8/35	22.9	10.4	39.2
Rietschen	3/11	27.3	6.0	61.0
Aquaculture company				
AC1	14/43	32.6	19.6	47.6
AC2	9/58	15.5	7.3	28.9
AC3	2/3	66.7	9.4	99.2

### 3.2 *Occurrence and molecular characterization of* Cryptosporidium spp.

For *Cryptosporidium*, the antigen test returned a positive result in 46 (44.2%; 95% CI: 34.5–54.3) and a negative result in 58 (55.8%; 95% CI: 45.7–65.5) out of 104 samples. While the PCR targeting the *SSU rRNA* gene yielded two (1.9%; 95% CI: 0.5–14.8) positive results, all samples tested negative at the *gp60* gene.

Both sequences were identical to each other, exhibiting 100% identity and query coverage with a *Cryptosporidium* sp. skunk genotype previously identified in an eastern grey squirrel (*Sciurus carolinensis*) in Italy (GenBank: MF411075). The sequences were deposited in GenBank under accession numbers PQ521012 and PX103175.

No significant associations were detected between *Cryptosporidium* positivity and any of the tested parameters: Year (*χ*^2^ = 1.438, *df* = 2, *p* = 0.487), location (*χ*^2^ = 2.676, *df* = 5, *p* = 0.750), aquaculture company (*χ*^2^ = 0.108, *df* = 2, *p* = 0.948), water supply (*p* = 1, OR = Inf, 95% CI: 0.005–Inf), sex (*χ*^2^ = 0.636, *df* = 1, *p* = 0.425) or age (*χ*^2^ = 1.762, *df* = 1, *p* = 0.184) (Fig. 1; Table C.2).

## 4 Discussion

The present study identifies raccoons in Eastern Saxon aquaculture as potential carriers of zoonotic *Giardia duodenalis* (Assemblage BIV) and *Cryptosporidium* (skunk genotype). These findings highlight a critical, understudied interface for waterborne pathogen transmission within the One Health framework, particularly in open pond systems where wildlife and commercial food production directly interact.

While *G. duodenalis* has been documented in raccoons within both their native North American range [39] and introduced European populations [20], molecular characterization of the assemblages involved remains limited. The present study provides the first report of sub-assemblage BIV in raccoons from Saxony and represents only the second report of this genotype in the species globally [20]. The observed prevalence of 24% aligns closely with the 27% previously reported in raccoons from Luxembourg and other German federal states, such as Baden-Württemberg and Hesse [20]. Thus, this zoonotic genotype seems to be stably established within Central European raccoon populations, and is of particular interest due to its low host specificity and zoonotic potential, having been identified in various German animal populations, including raccoons [20], [40], [41].

The significant association between *Giardia* positivity and specific locations suggests that local environmental factors are decisive in pathogen dynamics. These spatial variations indicate that site-specific conditions—such as raccoon population density, pond connectivity, or management practices (e.g., feeding regimes and water flow)—influence the local pathogen load. Notably, the varying prevalence rates between the responsible aquaculture companies may reflect differences in structural pond layouts or predator management, potentially facilitating raccoon-water interactions. Although the varying sample sizes across sites require a cautious interpretation, these findings underscore that zoonotic risk is heterogeneously distributed across the landscape. Thus, rather than applying generalized measures, there is a need for tailored, site-specific prevention strategies which focus on the interface between pond management and wildlife ecology.

*Cryptosporidium* skunk genotype (*C*SkG) was first identified in a striped skunk (*Mephitis mephitis*) from the USA [42], and has since been repeatedly reported in raccoons, including in Saxony [19]. It is hypothesized that *C*SkG was introduced to Europe via hosts native to North America [43], such as raccoons. Its recent detection in European badgers (*Meles meles*) in Poland [44], further suggests transmission between introduced and native species. Having also been detected in humans [45], *C*SkG is zoonotically relevant, raising concerns that raccoons and other wildlife could serve as sources of human infection, particularly in regions with close interaction with aquatic environments. These findings highlight the need for targeted management strategies to limit cross-species pathogen transmission and safeguard native populations. Given its zoonotic potential, continued monitoring—especially in aquaculture zones—is crucial for effective risk management.

The low PCR confirmation rate (2%) compared to the higher antigen positivity (24%) suggests that rapid tests should be interpreted with caution in wildlife studies. This discrepancy likely results from low oocyst shedding, potential cross-reactivity of the antigen assay with the raccoon's omnivorous diet, or the presence of PCR inhibitors in fecal matter.

As semi-aquatic mammals, raccoons frequently interact with water bodies [16], [46], potentially disseminating pathogens via feces through hydrological networks. The proximity of sampling sites to spring water catchment areas raises public health concerns regarding the contamination of drinking water supplies.

Beyond the water-borne route, the role of fish as mechanical vectors or reservoirs for these pathogens is of increasing concern. While this role remains a subject of debate, zoonotic *G. duodenalis* (including sub-assemblage BIV) and *Cryptosporidium* spp. have been identified in various freshwater species, such as rainbow trout and Cyprinidae [47], [48], [49], [50]. In the open pond systems characteristic of this region [4], [5], the lack of physical barriers allows raccoons to act as a recurring source of contamination. Given the documented cases of human giardiasis associated with raw or inadequately processed fish [51], [52], the growing interest in modern regional interpretations of local species—such as carpaccio or sashimi—significantly elevates the risk of human exposure at this wildlife-aquaculture interface. Importantly, current safety standards may be insufficient to mitigate these risks. Regulation (EC) No 853/2004 mandates freezing at −20 °C for 24 h to inactivate nematodes [53]. However, these protocols do not account for the fact that protozoan (oo)cysts can remain infectious even under such conditions [54], leaving a critical biosafety gap in food management. Furthermore, the transfer of fish from these ponds to other water bodies for stocking or further aquaculture may facilitate the anthropogenic spread of pathogens far beyond the initial study area, pointing to the need for integrated invasive species monitoring.

The present study reinforces the importance of the One Health approach, which acknowledges the profound interconnectedness of human, animal, and environmental health. The detection of zoonotic pathogens like *Giardia* and *Cryptosporidium* in raccoons—particularly within aquaculture zones—identifies a critical interface for disease transmission between wildlife, domestic animals, and humans.

The semi-aquatic lifestyle and high population density of raccoons make them potential bioindicators for monitoring pathogen loads in aquatic environments. Monitoring these populations may provide a more comprehensive overview of environmental contamination than sporadic sampling of water or fish. Aligning zoonotic surveillance with existing management frameworks, such as EU Regulation 1143/2014 [22], could address both biodiversity conservation and public health objectives. In practice, this could include the implementation of specialized fencing systems to exclude raccoons from sensitive aquaculture facilities and the intensification of predator control in high-risk zones to reduce environmental pathogen shedding. Furthermore, in light of the potential mechanical vector role of fish, food safety protocols for freshwater products from open systems should be re-evaluated to ensure they effectively inactivate protozoan (oo)cysts.

While this study provides important insights, the diagnostic workflow and sampling approach present certain limitations. The use of antigen screening as a prerequisite for PCR likely prioritized samples with higher pathogen loads, potentially omitting low-level infections. Although performing molecular analysis on all samples collected would have provided additional validation, this two-step screening approach was chosen due to budgetary and logistical constraints. Furthermore, the marked discrepancy between antigen positivity and molecular confirmation—particularly for *Cryptosporidium*—suggests that off-label use of rapid tests in wildlife should be interpreted with care. Finally, while opportunistic sampling of culled animals provides a valuable cross-sectional insight, it introduces a potential sampling bias as the health status of hunted individuals may not perfectly reflect natural population dynamics. Despite these methodological constraints, this research provides the first molecular evidence of zoonotic protozoa in raccoons within this specific aquaculture context. While direct environmental sampling was beyond the scope of this survey, our findings establish a vital foundation for future studies—which should incorporate water monitoring data to precisely map transmission pathways—as well as for integrated One Health strategies and invasive species management in water-rich production landscapes.

## 5 Conclusion

This study identifies invasive raccoons in aquaculture zones as potential reservoirs for zoonotic *Giardia duodenalis* (BIV) and *Cryptosporidium* (skunk genotype). The significant association between pathogen occurrence and specific locations underscores the need for integrated surveillance within the One Health framework. To mitigate zoonotic risks in these water-rich ecosystems, routine pathogen monitoring of invasive wildlife should be implemented as a standard procedure in aquaculture management. Aligning consistent screening with enhanced food safety protocols and predator control is essential to safeguard public health and ensure the biosafety of freshwater food production at the wildlife–aquaculture interface.

## CRediT authorship contribution statement

**Zoё Tess Lara Lindhorst:** Writing – original draft, Visualization, Methodology, Investigation, Formal analysis, Data curation. **Maria Sophia Unterköfler:** Writing – review & editing, Formal analysis. **Piotr Solarczyk:** Writing – review & editing, Resources. **Michael Striese:** Writing – review & editing, Resources. **Diana Jeschke:** Writing – review & editing, Resources. **Elisabeth Striese:** Writing – review & editing, Resources. **Hermann Ansorge:** Writing – review & editing, Resources. **Hans-Peter Fuehrer:** Writing – review & editing, Supervision, Project administration, Methodology, Conceptualization. **Mike Heddergott:** Writing – review & editing, Visualization, Supervision, Resources, Conceptualization.

## Funding

This research did not receive any specific grant from funding agencies in the public, commercial, or not-for-profit sectors.

## Declaration of competing interest

The authors declare a potential conflict of interest: MegaCor (Hohenems, Austria) provided the rapid immunochromatographic assays at a discounted rate. In exchange, the resulting data were shared with the manufacturer for further internal use.

## Data Availability

Data will be made available on request.
