# Supplementary material for: Detection of zoonotic protozoa in raccoons (Procyon lotor) from aquaculture zones in Saxony (Germany): One health perspective
Source: One Health. 2026 Jun 13;23:101477. doi: 10.1016/j.onehlt.2026.101477 (PMC13292377; doi:10.1016/j.onehlt.2026.101477)
Supplement: Supplementary file 1 — Supplementary material 1 [file mmc1.docx]

**Table A.1** PCR protocols and cycling conditions used for *Giardia duodenalis* in the present study

| **Target organism (genetic marker)** | **Mastermix protocol** | | | **Cycling Conditions** | | |
| --- | --- | --- | --- | --- | --- | --- |
|  | **Reagents** | **Unit** | **Quantity (µl) per tube** | **Temperature (°C)** | **Time** | **Step** |
| ***gdh*** |  |  |  | **Nest 1** |  |  |
|  | H_2_O |  | 30.35 | 94 | 2’ | Initial denaturation |
|  | green reaction buffer | 5 x | 10 | 94 | 45’’ | 40 x |
|  | dNTPs | 25 mM | 0.4 | 52 | 45’’ |  |
|  | TagPolymerase (GoTaq) | 5 u/µl | 0.25 | 72 | 1’ |  |
|  | Primer Forward | 100 pmol/µl | 2 | 72 | 5’ | Final extension |
|  | Primer Reverse | 100 pmol/µl | 2 | 15 | ~ | Storage |
|  | Template | 5 µl | 5 | **Nest 2** |  |  |
|  |  |  |  | 94 | 2’ | Initial denaturation |
|  |  |  |  | 94 | 45’’ | 35 x |
|  |  |  |  | 62 | 45’’ |  |
|  |  |  |  | 72 | 1’ |  |
|  |  |  |  | 72 | 5’ | Final extension |
|  |  |  |  | 15 | ~ | Storage |
| ***tpi*** |  |  |  | **Nest 1** |  |  |
|  | H_2_O |  | 30.35 | 94 | 2’ | Initial denaturation |
|  | green reaction buffer | 5 x | 10 | 94 | 45’’ | 40 x |
|  | dNTPs | 25 mM | 0.4 | 52 | 45’’ |  |
|  | TagPolymerase (GoTaq) | 5 u/µl | 0.25 | 72 | 1’ |  |
|  | Primer Forward | 100 pmol/µl | 2 | 72 | 5’ | Final extension |
|  | Primer Reverse | 100 pmol/µl | 2 | 15 | ~ | Storage |
|  | Template | 5 µl | 5 | **Nest 2** |  |  |
|  |  |  |  | 94 | 2’ | Initial denaturation |
|  |  |  |  | 94 | 45’’ | 35 x |
|  |  |  |  | 62 | 45’’ |  |
|  |  |  |  | 72 | 1’ |  |
|  |  |  |  | 72 | 5’ | Final extension |
|  |  |  |  | 15 | ~ | Storage |

**Table A.1 (continuation)** PCR protocols and cycling conditions used for *Giardia duodenalis* in the present study

| **Target organism (genetic marker)** | **Mastermix protocol** | | | **Cycling Conditions** | | |
| --- | --- | --- | --- | --- | --- | --- |
|  | **Reagents** | **Unit** | **Quantity (µl) per tube** | **Temperature (°C)** | **Time** | **Step** |
| ***bg*** |  |  |  | **Nest 1** |  |  |
|  | H_2_O |  | 30.35 | 94 | 2’ | Initial denaturation |
|  | green reaction buffer | 5 x | 10 | 94 | 45’’ | 40 x |
|  | dNTPs | 25 mM | 0.4 | 52 | 45’’ |  |
|  | TagPolymerase (GoTaq) | 5 u/µl | 0.25 | 72 | 1’ |  |
|  | Primer Forward | 100 pmol/µl | 2 | 72 | 5’ | Final extension |
|  | Primer Reverse | 100 pmol/µl | 2 | 15 | ~ | Storage |
|  | Template | 5 µl | 5 | **Nest 2** |  |  |
|  |  |  |  | 94 | 2’ | Initial denaturation |
|  |  |  |  | 94 | 45’’ | 35 x |
|  |  |  |  | 62 | 45’’ |  |
|  |  |  |  | 72 | 1’ |  |
|  |  |  |  | 72 | 5’ | Final extension |
|  |  |  |  | 15 | ~ | Storage |

**Table A.2** PCR protocols and cycling conditions used for *Cryptosporidium* spp. in the present study

| **Target organism (genetic marker)** | **Mastermix protocol** | | | **Cycling Conditions** | | | |
| --- | --- | --- | --- | --- | --- | --- | --- |
|  | **Reagents** | **Unit** | **Quantity (µl) per tube** | **Temperature (°C)** | **Time** | **Step** | |
| **18S *SSU rRNA*** |  |  |  | **Nest 1** |  |  |  |
|  | H_2_O |  | 30.35 | 94 | 2’ | Initial denaturation |  |
|  | green reaction buffer | 5 x | 10 | 94 | 45’’ | 40 x |  |
|  | dNTPs | 25 mM | 0.4 | 52 | 45’’ |  |  |
|  | TagPolymerase (GoTaq) | 5 u/µl | 0.25 | 72 | 1’ |  |  |
|  | Primer Forward | 100 pmol/µl | 2 | 72 | 5’ | Final extension |  |
|  | Primer Reverse | 100 pmol/µl | 2 | 15 | ~ | Storage |  |
|  | Template | 5 µl | 5 | **Nest 2** |  |  |  |
|  |  |  |  | 94 | 2’ | Initial denaturation |  |
|  |  |  |  | 94 | 45’’ | 35 x |  |
|  |  |  |  | 62 | 45’’ |  |  |
|  |  |  |  | 72 | 1’ |  |  |
|  |  |  |  | 72 | 5’ | Final extension |  |
|  |  |  |  | 15 | ~ | Storage |  |
| ***gp60*** |  |  |  | **Nest 1** |  |  |  |
|  | H_2_O |  | 30.35 | 94 | 2’ | Initial denaturation |  |
|  | green reaction buffer | 5 x | 10 | 94 | 45’’ | 40 x |  |
|  | dNTPs | 25 mM | 0.4 | 52 | 45’’ |  |  |
|  | TagPolymerase (GoTaq) | 5 u/µl | 0.25 | 72 | 1’ |  |  |
|  | Primer Forward | 100 pmol/µl | 2 | 72 | 5’ | Final extension |  |
|  | Primer Reverse | 100 pmol/µl | 2 | 15 | ~ | Storage |  |
|  | Template | 5 µl | 5 | **Nest 2** |  |  |  |
|  |  |  |  | 94 | 2’ | Initial denaturation |  |
|  |  |  |  | 94 | 45’’ | 35 x |  |
|  |  |  |  | 62 | 45’’ |  |  |
|  |  |  |  | 72 | 1’ |  |  |
|  |  |  |  | 72 | 5’ | Final extension |  |
|  |  |  |  | 15 | ~ | Storage |  |
