# Supplementary material for: Detection of zoonotic protozoa in raccoons (Procyon lotor) from aquaculture zones in Saxony (Germany): One health perspective
Source: One Health. 2026 Jun 13;23:101477. doi: 10.1016/j.onehlt.2026.101477 (PMC13292377; doi:10.1016/j.onehlt.2026.101477)
Supplement: Supplementary file 2 — Supplementary material 2 [file mmc2.docx]

**Table B.1** Assemblages and sub-assemblages used as references for the phylogenetic analysis of *G. duodenalis* in raccoons from Saxony in the present study. The list of references was obtained from Sprong et al. [1]

* Accession number with 100% identity and query coverage to original reference L02120 chosen, since no host available in original manuscript.

** Not the original publication cited, since none was available.

| **(Sub-)Assemblage** | **Reference** |
| --- | --- |
| AI | GU564275 [2] * |
| AII | U57897 [3] |
| AIII | DQ650648 [4] |
| BIII | AF069561 [5] |
| BIV | AF069560 [5] |
| C | AY228641 [6] |
| D | DQ246216 [1] ** |
| E | AY228645 [6] |
| F | AF069558 [5] |
| G | EU781013 [7] |
| H | None available |

**References**

[1] H. Sprong, S.M. Cacciò, J.W.B. van der Giessen, on behalf of the Z. network and Partners, Identification of Zoonotic Genotypes of Giardia duodenalis, PLoS Negl Trop Dis 3 (2009) e558. https://doi.org/10.1371/journal.pntd.0000558.

[2] M.R. Mowatt, E.C. Weinbach, T.C. Howard, T.E. Nash, Complementation of an *Escherichia coli* Glycolysis Mutant by *Giardia lamblia* Triosephosphate Isomerase, Exp Parasitol 78 (1994) 85–92. https://doi.org/10.1006/expr.1994.1008.

[3] A.C. Baruch, J. Isaac-Renton, R.D. Adam, The Molecular Epidemiology of Giardia lamblia: A Sequence-Based Approach, J Infect Dis 174 (1996) 233–236. https://doi.org/10.1093/infdis/174.1.233.

[4] M. Lalle, A.F. di Regalbono, L. Poppi, G. Nobili, D. Tonanzi, E. Pozio, S.M. Cacciò, A Novel Giardia duodenalis Assemblage A Subtype in Fallow Deer, J Parasitol 93 (2007) 426–428. https://doi.org/10.1645/GE-983R.1.

[5] P.T. Monis, R.H. Andrews, G. Mayrhofer, P.L. Ey, Molecular systematics of the parasitic protozoan Giardia intestinalis., Mol Biol Evol 16 (1999) 1135–1144. https://doi.org/10.1093/oxfordjournals.molbev.a026204.

[6] I.M. Sulaiman, R. Fayer, C. Bern, R.H. Gilman, J.M. Trout, P.M. Schantz, P. Das, A.A. Lal, L. Xiao, Triosephosphate Isomerase Gene Characterization and Potential Zoonotic Transmission of Giardia duodenalis, Emerg Infect Dis 9 (2003) 1444–1452. https://doi.org/10.3201/eid0911.030084.

[7] M. Lebbad, J.G. Mattsson, B. Christensson, B. Ljungström, A. Backhans, J.O. Andersson, S.G. Svärd, From mouse to moose: Multilocus genotyping of *Giardia* isolates from various animal species, Vet Parasitol 168 (2010) 231–239. https://doi.org/10.1016/j.vetpar.2009.11.003.
