# Supplementary material for: Detection of zoonotic protozoa in raccoons (Procyon lotor) from aquaculture zones in Saxony (Germany): One health perspective
Source: One Health. 2026 Jun 13;23:101477. doi: 10.1016/j.onehlt.2026.101477 (PMC13292377; doi:10.1016/j.onehlt.2026.101477)
Supplement: Supplementary file 3 — Supplementary material 3 [file mmc3.docx]

**Table C.1** Associations in *Giardia* positivity by host factors in raccoons from Saxony.

*Statistically significant (*p* < 0.05)

| **Category** | **n/N** | **Proportion (%)** | **95% CI Lower (%)** | **95% CI Upper (%)** |
| --- | --- | --- | --- | --- |
|  |  |  |  |  |
| **Location of death*** |  |  |  |  |
| Daubitz | 4/10 | 40.0 | 12.2 | 73.8 |
| Hammerstadt | 7/22 | 31.8 | 13.9 | 54.9 |
| Kreba West | 2/3 | 66.7 | 9.4 | 99.2 |
| Niederspree | 1/23 | 4.3 | 0.1 | 22.0 |
| Quolsdorf | 8/35 | 22.9 | 10.4 | 39.2 |
| Rietschen | 3/11 | 27.3 | 6.0 | 61.0 |
|  |  |  |  |  |
| **Aquaculture company*** |  |  |  |  |
| AC1 | 14/43 | 32.6 | 19.6 | 47.6 |
| AC2 | 9/58 | 15.5 | 7.3 | 28.9 |
| AC3 | 2/3 | 66.7 | 9.4 | 99.2 |
|  |  |  |  |  |
| **Year of death** |  |  |  |  |
| 2020 | 15/61 | 24.6 | 14.5 | 37.3 |
| 2021 | 1/3 | 33.3 | 0.8 | 90.6 |
| 2022 | 9/40 | 22.5 | 10.8 | 38.5 |
|  |  |  |  |  |
| **Water supply** |  |  |  |  |
| Weißer Schöps | 23/101 | 22.8 | 15.0 | 32.2 |
| Schwarzer Schöps | 2/3 | 66.7 | 9.4 | 99.2 |
|  |  |  |  |  |
| **Sex** |  |  |  |  |
| Male | 12/55 | 21.8 | 11.8 | 35.0 |
| Female | 13/49 | 26.5 | 14.9 | 41.1 |
|  |  |  |  |  |
| **Age** |  |  |  |  |
| Adult (> 1 year) | 20/71 | 28.2 | 18.1 | 40.1 |
| Juvenile (≤ 1 year) | 5/33 | 15.2 | 5.1 | 31.9 |

**Table C.2** Associations in *Cryptosporidium* positivity by host factors in raccoons from Saxony.

| **Category** | **n/N** | **Proportion (%)** | **95% CI Lower (%)** | **95% CI Upper (%)** |
| --- | --- | --- | --- | --- |
|  |  |  |  |  |
| **Location of death** |  |  |  |  |
| Daubitz | 0/10 | 0.0 | 0.0 | 30.8 |
| Hammerstadt | 1/22 | 4.5 | 0.1 | 22.8 |
| Kreba West | 0/3 | 0.0 | 0.0 | 70.8 |
| Niederspree | 1/23 | 4.3 | 0.1 | 21.9 |
| Quolsdorf | 0/35 | 0.0 | 0.0 | 10.0 |
| Rietschen | 0/11 | 0.0 | 0.0 | 28.5 |
|  |  |  |  |  |
| **Aquaculture company** |  |  |  |  |
| AC1 | 1/43 | 2.3 | 0.1 | 12.3 |
| AC2 | 1/58 | 1.7 | 0.0 | 9.2 |
| AC3 | 0/3 | 0.0 | 0.0 | 70.8 |
|  |  |  |  |  |
| **Year of death** |  |  |  |  |
| 2020 | 2/61 | 3.3 | 0.4 | 11.3 |
| 2021 | 0/3 | 0.0 | 0.0 | 70.8 |
| 2022 | 0/40 | 0.0 | 0.0 | 8.8 |
|  |  |  |  |  |
| **Water supply** |  |  |  |  |
| Weißer Schöps | 2/101 | 2.0 | 0.2 | 7.0 |
| Schwarzer Schöps | 0/3 | 0.0 | 0.0 | 70.8 |
|  |  |  |  |  |
| **Sex** |  |  |  |  |
| Male | 0/55 | 0.0 | 0.0 | 6.5 |
| Female | 2/49 | 4.1 | 0.5 | 14.0 |
|  |  |  |  |  |
| **Age** |  |  |  |  |
| Adult (> 1 year) | 0/71 | 0.0 | 0.0 | 5.1 |
| Juvenile (≤ 1 year) | 2/33 | 6.1 | 0.7 | 20.2 |
